# The Prevalence and Predictors of Atrioventricular Blocks in Syrian Patients Reporting to the Emergency Department During the Ongoing Conflict: A Cross‐Sectional Study

**DOI:** 10.1111/jce.16578

**Published:** 2025-01-13

**Authors:** Ibrahim Antoun, Alkassem Alkhayer, Aref Jalal Eldin, Alamer Alkhayer, Khaled Yazji, Riyaz Somani, G. André Ng, Mustafa Zakkar

**Affiliations:** ^1^ Faculty of Medicine University of Aleppo Aleppo Syria; ^2^ Department of Medicine University of Tishreen's Hospital Latakia Syria; ^3^ Department of Cardiology The View Hospital Qatar Qatar; ^4^ Faculty of Medicine University of Damascus Damascus Syria; ^5^ Department of Cardiovascular Sciences University of Leicester Leicester UK; ^6^ Department of Cardiology University Hospitals of Leicester NHS Trust, Glenfield Hospital Leicester UK; ^7^ NIHR Leicester Biomedical Research Centre Leicester UK; ^8^ Department of Cardiac Surgery University Hospitals of Leicester NHS Trust, Glenfield Hospital Leicester UK

**Keywords:** conflict, ECG, heart block, screening, Syria

## Abstract

**Background:**

Atrioventricular block (AVB) is a cardiac conduction disorder that can lead to significant clinical outcomes, particularly in resource‐limited and conflict‐affected regions. In Syria, healthcare infrastructure has been severely impacted by ongoing conflict, potentially affecting the prevalence and management of AVB.

**Methods:**

We conducted a cross‐sectional study at Tishreen University Hospital in Latakia, Syria, with patients > 40 who presented to the emergency department (ED) from June 1 to August 1, 2024. Routine 12‐lead ECGs were performed, with AVB diagnoses confirmed by two independent cardiology consultants. Exclusion criteria included pre‐existing AVB, pacemaker presence, hemodynamic instability, and ECG diagnostic discrepancies. Patient demographics and comorbidities were assessed, and logistic regression analyses identified predictors of AVB.

**Results:**

The final analysis included 692 patients, of which AVB was detected in 7% of the cohort. Patients with AVB were significantly older (median age 76 vs. 54 years, *p* < 0.001) and had higher rates of ischemic heart disease (IHD) (62% vs. 14%, *p* < 0.001) and diabetes (56% vs. 21%, *p* = 0.01). Logistic regression showed older age (odds ratio [OR]: 2.8, 95% confidence interval [CI]: 1.6−5.6, *p* < 0.001), IHD (OR: 1.9, 95% CI: 1.4−4.5, *p* < 0.001), and DM (OR: 3.9, 95% CI: 2.9−8.3, *p* < 0.001) were independently associated with AVB.

**Conclusion:**

AVB prevalence in the Syrian ED setting is high, with age, IHD, and diabetes as significant predictors. Routine ECG screening in EDs may facilitate early AVB detection in at‐risk populations, especially in conflict‐affected regions with limited healthcare resources. This approach could improve outcomes by enabling timely intervention in high‐risk patients.

## 1 Introduction

Atrioventricular block (AVB) is a significant cardiac conduction disorder characterized by impaired transmission of electrical impulses from the atria to the ventricles. This condition can manifest in various degrees, ranging from first‐degree block, which is often asymptomatic, to complete heart block (CHB), which can lead to severe clinical consequences such as syncope or sudden cardiac death [1, 2]. Acquired AVB forms can arise from conditions such as myocardial infarction, cardiac surgery, and inflammatory diseases [3, 4]. In the context of acute myocardial infarction, for instance, high‐degree AVB is frequently observed and is associated with increased morbidity and mortality [5, 6].

Furthermore, factors such as age, sex, and comorbidities like diabetes and hypertension have been identified as significant predictors of AVB development [7]. The prevalence of AVB varies across different populations and clinical settings, making it crucial to understand its occurrence in specific countries, especially low‐to‐middle‐income countries. Syria has been suffering from a conflict since 2011. It has been deprived of healthcare funding and resources, particularly exacerbated during the cholera and COVID‐19 outbreaks [8, 9]. Therefore, less than 50% of its hospitals operate as usual, with more than half its healthcare workforce forced to leave the country due to conflict [10]. In the Syrian context, the prevalence of AVB may be influenced by unique demographic and health factors, including the impact of ongoing conflict on healthcare access and the management of chronic diseases. Studies have shown that high‐degree AVB is particularly prevalent in populations with underlying cardiac conditions, such as hypertrophic cardiomyopathy and ischemic heart disease (IHD) [11]. Routine screening in emergency departments (ED) could facilitate the early identification of AVB, allowing for timely intervention and management. This is critical, given the potential for rapid deterioration in patients with CHB [12, 13].

Moreover, the implementation of electrocardiographic screening protocols in emergency settings has been advocated to improve the detection rates of AVB, especially in asymptomatic patients who may be at risk due to underlying conditions [14, 15]. This approach is particularly relevant in regions like Syria, where healthcare resources may be limited, and the burden of cardiovascular diseases is rising. Understanding the prevalence and risk factors associated with AVB in this population will provide valuable insights for healthcare providers and policymakers, ultimately enhancing patient outcomes through targeted screening and management strategies. This study aimed to assess the prevalence of AVB in Syrian patients visiting ED during the ongoing conflict.

## 2 Methods

### 2.1 Study Design and Data Collection

This single‐center cross‐sectional observational study was conducted at Tishreen's University Hospital, Latakia, Syria. It is a large teaching hospital and tertiary care center with around 860 beds. On average, the hospital provides free healthcare to approximately 50 000−60 000 inpatients yearly, with an even more significant number of outpatients seeking care in various medical departments. The ED has around 50 beds across different units, including triage, critical care, and observation. The study included patients over 40 years old reporting the ED between June 1, 2024, and August 1, 2024. A 12‐lead ECG was conducted routinely regardless of the presenting complaint. Two general cardiology consultants blindly reviewed ECGs. We excluded patients with known AVB, patients with pacemakers, patients with a critical condition or hemodynamic instability, and patients with discrepancies in ECG diagnosis between the two cardiology consultants. ED and medical clinical charts were examined for patients' demographics. The research reported in this article adhered to the Declaration of Helsinki. The project was conducted as part of an audit approved by the hospital board and involved prospective analysis of anonymized collected data. The reporting of this observational study followed the Strengthening the Reporting of Observational Studies in Epidemiology (STROBE) guidelines [16].

### 2.2 Study Outcomes

The study's primary outcomes included AVB on ECG screening. A secondary analysis explored the predictors of AVB.

### 2.3 Statistical Analysis

Continuous variables are expressed as median and interquartile range (IQR). Categorical variables are expressed as counts and percentages (%). Pearson's *χ* ^2^ or Fisher's exact test was used for categorical variables between groups. Students' *t*‐tests and Kruskal−Wallis tests were used to compare continuous variables between the groups depending on the normality of the distribution. We used logistic regression to examine the relationship between demographics and the presence of AVB. Our multivariable model was constructed a priori and included demographics that are statistically significant in the univariable model. A two‐sided *p* < 0.05 was considered statistically significant. Statistical analysis was performed using GraphPad Prism V10.3 for Mac (San Diego, CA, USA).

## 3 Results

### 3.1 Patients Characteristics and Primary Outcomes

**Figure 1:**
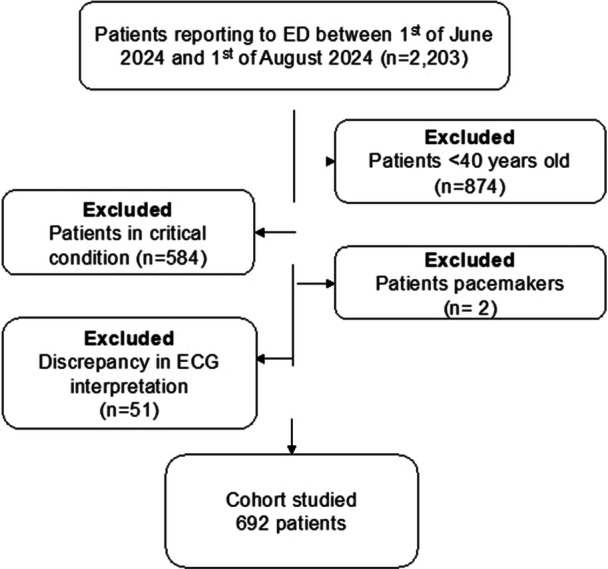
Patients' selection criteria. ECG, electrocardiogram; ED, emergency department.

After applying the selection criteria in Figure 1, 692 patients were included in the final analysis. Of these, 45 had AVB proven on the 12‐lead ECG (7%). None of the discrepancies in the two cardiologists' reports involved AVB patients. Table 1 demonstrates the demographics of the study cohort. Males comprised 52% of the cohort, with a median age of 60 (IQR: 54−67 years). Compared to the rest of the patients, AVB patients were older (76 vs. 54 years; *p* < 0.001), had more IHD patients (62% vs. 14%; *p* < 0.01) and more diabetes mellitus (DM) patients (56% vs. 21%; *p* = 0.01).

**Table 1:** Demographics and characteristics of patients stratified by 12‐lead electrocardiogram results.

	Overall (*n* = 692)	AVB (*n* = 45)	No AVB (647)	*p* value
Demographics, *n* (%) or median (IQR)				
Age (years)	60 (54−67)	76 (74−81)	54 (40−60)	< 0.001
Male	361 (52%)	22 (49%)	339 (52%)	0.45
Cardiovascular comorbidities, *n* (%)				
Hypertension	232 (34%)	16 (36%)	216 (33%)	0.48
Ischemic heart disease	119 (17%)	28 (62%)	91 (14%)	< 0.001
Diabetes mellitus	158 (23%)	25 (56%)	133 (21%)	0.01
Cerebrovascular disease	121 (19%)	11 (22%)	110 (19%)	0.57
Congestive heart failure	131 (19%)	9 (20%)	122 (19%)	0.12
Other comorbidities, *n* (%)				
Anemia	112 (16%)	10 (22%)	102 (16%)	0.62
Active malignancy	51 (7%)	2 (4%)	49 (8%)	0.22
Chronic liver failure	37 (5%)	5 (11%)	32 (5%)	0.11
Chronic lung disease	74 (11%)	3 (7%)	71 (11%)	0.89
Chronic kidney failure	75 (11%)	5 (11%)	70 (11%)	0.91
Thyroid disease	30 (4%)	3 (7%)	27 (4%)	0.44
Electrolytes and medications, *n* (%)				
Rate limiting drugs	181 (26%)	15 (33%)	166 (26%)	0.06
Hypokalaemia^a^	23 (10%)	4 (14%)	19 (10%)	0.51
Hypocalcaemia^a^	25 (11%)	3 (10%)	22 (11%)	0.96
Hypomagnesaemia^a^	14 (6%)	2 (7%)	12 (6%)	0.85

Abbreviation: AVB, atrioventricular block.

^a^ Only conducted in 229/692.

**Figure 2:**
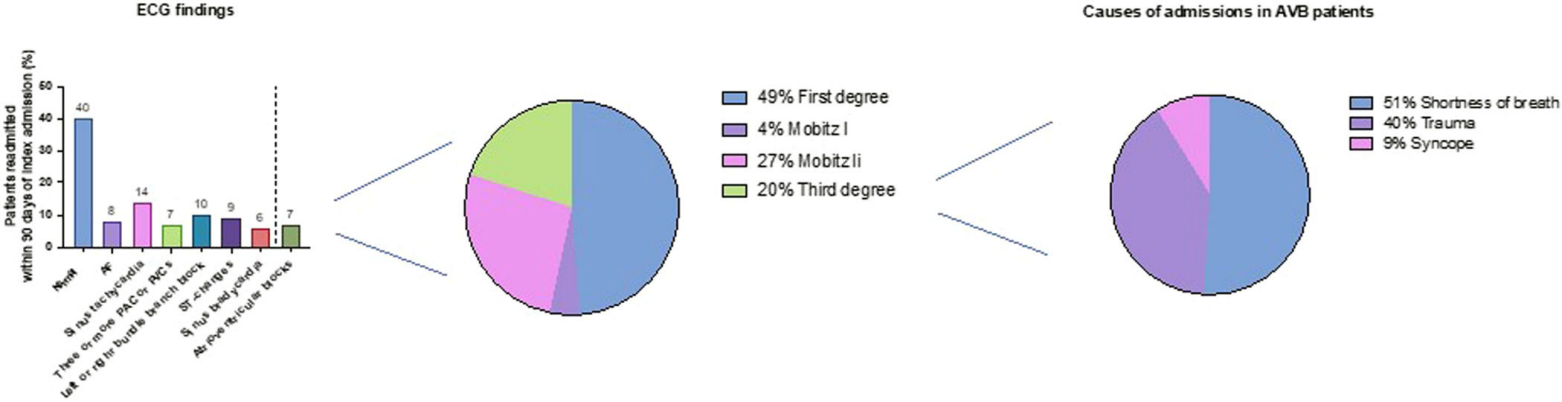
Admissions etiology and electrocardiogram results. AF, atrial fibrillation; AVB, atrioventricular block; ECG, electrocardiogram; PAC, premature atrial contraction; PVC, premature ventricular contraction.

The ECG findings and patients' presenting complaints are shown in Figure 2. ECG was normal in 279 (40%). The most common ECG abnormality was sinus tachycardia in 95 patients (14%), followed by frequent atrial or ventricular ectopy in 47 patients (7%) and bundle branch block in 66 patients (10%). The most common presenting complaints were trauma in 304 patients (44%), followed by respiratory complaints in 84 patients (12%) and chest pain/syncope in 49 (7%). In patients with AVB, the most common presenting complaint was shortness of breath in 23 patients (51%), followed by trauma in 18 patients (40%) and syncope in 4 patients (9%). Of the AVB patients, 49% were first‐degree, 4% were Mobitz I, 27% were Mobitz II, and 20% were third‐degree blocks. The last two were referred to another nearby center to have pacemakers implanted.

### 3.2 Secondary Outcomes

Logistic regression models are demonstrated in Table 2. Univariable regression showed that older age (odds ratio [OR]: 2.6, 95% confidence interval [CI]: 1.4−4.9, *p* = 0.02), IHD (OR: 2.9, 95% CI: 1.8−5.4, *p* < 0.001), and DM (OR: 3, 95% CI: 1.8−5.1, *p* = 0.004) were associated with the presence of AVB. The multivariable logistic regression model demonstrated that older age (OR: 2.8, 95% CI: 1.6−5.6, *p* < 0.001), IHD (OR: 1.9, 95% CI: 1.4−4.5, *p* < 0.001), and DM (OR: 3.9, 95% CI: 2.9−8.3, *p* < 0.001) were independently associated with AVB.

**Table 2:** Logistic regression model regarding predictors of atrioventricular block in the study.

Variables	Univariable analysis		Multivariable analysis	
	OR (95% CI)	*p* value	OR (95% CI)	*p* value
Age (per year increase)	2.6 (1.4−4.9)	0.01	2.8 (1.6−5.5)	< 0.001
Sex (female compared to male)	1 (0.9−1)	0.94		
Diabetes mellitus (yes vs. no)	3 (1.8−5.1)	< 0.001	3.9 (2.9−8.3)	< 0.001
Ischemic heart disease (yes vs. no)	2.9 (1.8−5.4)	< 0.001	1.9 (1.4−4.5)	< 0.001
Heart failure (yes vs. no)	1.2 (0.7−2.4)	0.12		
Cerebrovascular event (yes vs. no)	1.1 (0.6−2.9)	0.77		
Hypertension (yes vs. no)	1.1 (0.6−2.6)	0.69		
Chronic lung disease (yes vs. no)	1 (0.8−1.1)	0.89		
Thyroid disease (yes vs. no)	1.1 (0.4−3.4)	0.75		
Anemia (yes vs. no)	1 (0.9−1)	0.22		
Active malignancy (yes vs. no)	1 (0.8−1.2)	0.78		
Chronic liver failure (yes vs. no)	0.9 (0.7−1.3)	0.65		
Chronic lung disease (yes vs. no)	1 (0.9−1)	0.95		
Chronic kidney failure (yes vs. no)	1.1 (0.8−1.1)	0.88		
Rate limiting drugs (yes vs. no)	1.2 (0.7−1.7)	0.11		
Hypokalaemia (yes vs. no)	0.8 (0.4−1.8)	0.48		
Hypocalcaemia (yes vs. no)	0.9 (0.3−2.2)	0.33		
Hypomagnesaemia (yes vs. no)	1.1 (0.7−1.7)	0.25		

Abbreviations: CI, confidence interval; OR, odds ratio.

## 4 Discussion

There has been a recent effort to unmask arrhythmias in Syria during the conflict [17, 18, 19, 20]. This is the first study to address the prevalence of AVB in Syria and the Middle East. The observed AVB prevalence was 7%, highlighting a substantial occurrence rate within this context. Our AVB rate supersedes the Chinese nationwide AVB rate of 0.6% [21]. This can be partially attributed to the Syrian conflict, which has been ongoing since 2011 and has massively affected health infrastructure. It resulted in a high turnover of skilled staff and inadequate nurses and allied health professionals [22]. Only half of the country's hospitals and primary healthcare centers are fully functional [22]. Although there was no data before the conflict for comparison, the current data is likely to reflect the current state of play throughout this war‐torn country. This included reduced access to preventive healthcare and a higher burden of cardiovascular risk factors, psychological stress, and disruptions in healthcare infrastructure, which likely contributed to our increased AVB pickup rate. Our study adds to the existing literature by identifying key predictors associated with AVB, particularly in a population affected by conflict and resource scarcity.

### 4.1 Factors Associated With AVB

Our results align with previous findings that age, IHD, and DM are significant predictors of AVB [7, 21]. Age was notably associated with a higher risk of AVB (OR: 2.8, *p* < 0.001), which is expected, as AVB incidence tends to increase with age due to degenerative changes in the conduction system [23]. The association between IHD and AVB is well‐documented, often explained by ischemia‐related damage to the atrioventricular conduction pathway. In our study, IHD had an OR of 1.9 in the multivariable model, reflecting the substantial risk it poses. Similarly, DM showed a strong association with AVB (OR: 3.9), likely related to the effects of hyperglycemia on vascular health and microvascular complications that can affect the heart's conduction system.

### 4.2 Impact of Conflict on AVB Prevalence and Healthcare Access

The unique demographic and healthcare conditions in Syria likely influence the prevalence and characteristics of AVB in this population. The ongoing conflict has led to a deterioration of healthcare services, with limited resources for managing chronic conditions that can predispose individuals to AVB. Only a fraction of the hospitals remain fully functional, and a significant proportion of healthcare professionals have migrated, creating a gap in care [22, 24]. These factors may contribute to the observed prevalence of AVB and highlight the need for effective screening and management protocols in similar settings. Given the elevated cardiovascular disease burden in Syria, especially amid poor healthcare accessibility, identifying AVB in the ED setting is crucial to mitigate potential adverse outcomes in high‐risk patients.

### 4.3 Implications for Screening and Management

This study underscores the importance of implementing ECG screening protocols in resource‐limited emergency settings, where cardiovascular diseases may go undiagnosed until advanced stages. Routine ECGs can be a critical tool for early AVB detection, enabling timely intervention and reducing the risk of progression to higher degrees of AVB [21]. Given that AVB can often be asymptomatic, especially in the early stages, ECG screening can help capture cases that might otherwise remain unidentified. For Syrian healthcare providers and policymakers, prioritizing ECG screening in ED could significantly improve outcomes, particularly in older adults and those with comorbidities such as IHD and DM.

## 5 Limitations

Data collection was limited to a single tertiary care center in Latakia. This city was relatively less affected by the Syrian conflict than the other northern and eastern regions of Syria. Therefore, our results might not be generalizable to other centers/regions, given the significant heterogeneity in the quality and level of hospital supplies and staffing. Additionally, our analysis included only routinely collected data within the medical records and by the number of patients who presented to the hospital. Not all patients had routine blood tests, potentially missing potential risk factors for AVB.

## 6 Conclusion

There was a significant AVB prevalence in this conflict‐affected Syrian population, with age, IHD, and DM identified as key predictors. Given the limited healthcare resources and high cardiovascular burden in Syria, our findings underscore the need for targeted ECG screening in Syrian EDs to facilitate early AVB detection and management. Improving screening protocols in such settings could be instrumental in reducing the risk of complications and enhancing patient outcomes in populations facing healthcare access challenges.

## Author Contributions

I.A. designed the study, analyzed the data, and wrote the first draft of the manuscript. A.A. and A.J.E. managed data collection. A.S., A.A., M.Z., K.Y., G.A.N., and R.S. reviewed and edited the manuscript.

## Ethics Statement

The study was part of an audit approved by Tishreen's University Hospital's ethical board. The project was conducted as part of an audit approved by the hospital board and involved prospective analysis of anonymized collected data. Therefore, the institution's ethical board waived the need for consent.

## Conflicts of Interest

The authors declare no conflicts of interest.

## Data Availability

The data that support the findings of this study are available from the corresponding author upon reasonable request.
